# DFT-Based Studies on Carbon Adsorption on the wz-GaN Surfaces and the Influence of Point Defects on the Stability of the Diamond–GaN Interfaces

**DOI:** 10.3390/ma14216532

**Published:** 2021-10-29

**Authors:** Malgorzata Sznajder, Roman Hrytsak

**Affiliations:** 1 Institute of Physics, College of Natural Sciences, University of Rzeszow, Pigonia 1, 35-959 Rzeszow, Poland; rhrytsak@ur.edu.pl; 2 Institute of High Pressure Physics, Polish Academy of Sciences, Sokolowska 29/37, 01-142 Warsaw, Poland

**Keywords:** diamond crystal, gallium nitride (GaN), adsorption, heterojunction, interface structure, defects

## Abstract

Integration of diamond with GaN-based high-electron-mobility transistors improves thermal management, influencing the reliability, performance, and lifetime of GaN-based devices. The current GaN-on-diamond integration technology requires precise interface engineering and appropriate interfacial layers. In this respect, we performed first principles calculation on the stability of diamond–GaN interfaces in the framework of density functional theory. Initially, some stable adsorption sites of C atoms were found on the Ga- and N-terminated surfaces that enabled the creation of a flat carbon monolayer. Following this, a model of diamond–GaN heterojunction with the growth direction [111] was constructed based on carbon adsorption results on GaN{0001} surfaces. Finally, we demonstrate the ways of improving the energetic stability of diamond–GaN interfaces by means of certain reconstructions induced by substitutional dopants present in the topmost GaN substrate’s layer.

## 1. Introduction

III-nitride-based high-electron-mobility transistors (HEMTs), especially GaN-based HEMT devices, are in the focus of next-generation tetrahertz communications, radar detection systems, high-power RF applications, as well as massive production for the incoming 5G LTE base stations [1]. The wurtzite GaN semiconductor possesses excellent properties: a wide bandgap of 3.4 eV [2], a high intrinsic breakdown electric field (3 MV/cm [3]), a high-saturation electron velocity ($3\times 10^{7}$ cm/s) [4], as well as the ability to form a high-density, high-mobility two-dimensional electron gas achieved by appropriate bandgap engineering (e.g., induced at the AlGaN–GaN interface due to spontaneous and piezoelectric polarization). These excellent material properties enable the high switching speed of AlGaN/GaN HEMTs, leading to high-power densities. The maximum theoretical radio-frequency power devices for GaN-based HEMTs reported to date is 40 W/mm at 4 GHz [5]. As the output power density of GaN-based HEMT devices increases, the dissipation of the heat flux present in the channel becomes more and more important. Hence, thermal management plays a critical role in the performance, reliability, and lifetime of these devices. The same concerns innovative GaN-based light-emitting diode (LED) designs that are capable of operating at high current densities, offering at the same time a low thermal resistance [6,7,8].

Diamond in turn is the material with the highest thermal conductivity of 2000 W/m·K [9], a high saturation velocity [10] and carrier mobility [11], as well as a large indirect bandgap (5.5 eV) [12]. Therefore, the integration of GaN with a high thermal conductivity substrate such as diamond can improve the heat extraction from GaN-based HEMT and decrease the operating temperature. However, heterogeneous integration of GaN with diamond leading to the GaN-on-diamond architecture is still challenging. Up to now, three kinds of approaches to fabricate the GaN-on-diamond architecture have been demonstrated: (i) diamond growth on GaN [13,14,15,16], (ii) GaN growth on diamond [17,18,19], and (iii) GaN–diamond bonding technology [20,21,22,23]. In the first two approaches, a nucleation layer is formed at the initial stage of growth, resulting in a large number of defects and grain boundaries at the interface, which have a negative impact on the thermal performance of the GaN-on-diamond device due to the poor thermal conductivity [5]. In order to overcome this problem, a high-quality interfacial layer (e.g., ${\mathrm{SiN}}_{x}$, AlN) with optimized thickness is applied. In the third approach, both the diamond substrate and GaN-based HEMT structure can be prepared in parallel, and issues related to a large lattice and thermal expansion coefficient mismatch are reduced, as compared to direct heteroepitaxial growth of diamond on GaN, and vice versa. However, even the bonding process typically requires a bonding interfacial layer that usually exhibits poor thermal conductivity and leads to an increase in the effective thermal boundary resistance (${\mathrm{TBR}}_{\mathrm{eff}}$) of the fabricated GaN-on-diamond structure. This quantity, ${\mathrm{TBR}}_{\mathrm{eff}}$, should be minimized to achieve the optimal performance of the GaN-on-diamond device [24]. This can be realized by improving both the diamond substrate quality and interface engineering, including the choice, quality, and thickness of the interfacial layer material [13,25].

Hence, at this point, it is reasonable to supplement the existing experimental results with a theoretical analysis concerning diamond–GaN interfaces, with the focus on some reconstructions that can occur when species of different valencies (Ga, N, Si, C) create bonding at the interface. Since a wz-GaN crystal exhibits total macroscopic polarization, which is the sum of the spontaneous and the strain-induced (piezoelectric) polarization [2], such a built-in electric field in 〈0001〉 directions can influence the diamond–GaN interface. Note also that the piezoelectric part of polarization can be influenced by defects, e.g., lowered when the strain in the deposited/interfacial layers is reduced by the presence of defects. The present paper aims to demonstrate that certain reconstruction patterns involving substitutional atoms can improve the energetic stability of the diamond–GaN interfaces, as was observed in the case of diamond–AlN interfaces [26]. Since the proposed reconstruction patterns take place in the GaN substrate, our findings are closely related to GaN-on-diamond experimental approaches where the formation of carbide bonds [27] or a silicon–carbon–nitrogen layer between GaN and diamond [16] was reported.

In order to prepare a reliable model of the diamond–GaN interface, information about the carbon adsorption sites on GaN$\left\{000\bar{1}\right\}$ surfaces is necessary. Such studies have been performed so far mainly from the viewpoint of the reduction of carbon contamination during GaN growth by means of metalorganic vapor phase epitaxy (MOVPE). In particular, Kempisty et al. [28] studied the incorporation and interdiffusion of carbon in GaN$\left\{000\bar{1}\right\}$ surfaces reconstructed during typical MOVPE growth conditions (i.e., $\left(000\bar{1}\right)$ 3N-H, $\left(000\bar{1}\right){\mathrm{Ga}}_{\mathrm{ad}}\left(H3\right)$, (0001)${\mathrm{Ga}}_{\mathrm{ad}}\left(T4\right)$, and (0001)3Ga-H) and demonstrated the influence of surface states, as well as the role of the $N_{2}$ and $H_{2}$ carrier gases on carbon incorporation. Kusaba et al. [29] in turn performed sequential analysis concerning the adsorption of ${\mathrm{CH}}_{4}$ on GaN$\left\{000\bar{1}\right\}$ reconstructed surfaces during MOVPE (surface reconstruction, ${\mathrm{CH}}_{4}$ adsorption, and C incorporation) to explain the influence of the growth conditions and surface termination on the C concentration in GaN films. However, for the purpose of the present study, mainly the information about the carbon adsorption sites on clean GaN$\left\{000\bar{1}\right\}$ surfaces is necessary, in order to construct a general model of the diamond–GaN interface. Therefore, our results on carbon adsorption on both the (0001) and $\left(000\bar{1}\right)$ polar surfaces are supplied.

The present paper is organized as follows. Section 2 presents the details about the applied calculation method and model. Section 3 provides results concerning the adsorption process of carbon atoms on the GaN(0001) (Ga-terminated Ga-face) and GaN$\left(000\bar{1}\right)$ (N-terminated N-face) surfaces. The most energetically stable adsorption sites are found that justify the use of a certain diamond–GaN interface model in Section 3.1.2. Next, some reconstructions occurring in the topmost GaN substrate’s layer and involving certain substitutional dopants are proposed that ensure charge neutrality within the lateral cell at the diamond–GaN interfaces (Section 4.1). For this purpose, in Section 4.2, the heights of the migration energy barriers of certain point defects present in GaN bulk crystals are calculated. Finally, the energetic stability of the reconstructed diamond–GaN interfaces is analyzed in Section 4.3.

## 2. Methods and Models

Our calculations were performed by means of the density functional theory (DFT) using the Siesta code [30]. The generalized gradient approximation (GGA) was used to describe the electron exchange–correlation interactions, and in the case of C and H atoms, the Perdew–Burke–Ernzerhof (PBE) form of the exchange–correlation functional [31] was utilized. In the case of Ga and N atoms, the PBEJsJrHEG functional was applied. PBEJsJrHEG stands for the GGA-PBE functional with parameters β, μ fixed by the jellium surface, jellium response, and κ fixed by the Lieb–Oxford bound for the low-density limit of the homogeneous electron gas [31,32,33]. The Troullier–Martins-type [34] pseudopotentials were chosen to describe the electron ion–core interactions, and the basis functions had the following size: C–2s: DZ (double zeta), 2p: DZP (double zeta polarized); Ga–4s: TZ (triple zeta), 4p: TZ, 3d: SZ (single zeta); N–2s: TZ, 2p: TZP (triple zeta polarized); H–1s: DZ. A grid in real space was obtained using an equivalent plane wave cutoff of 400 Ry, while integration in the *k*-space was conducted employing (11×11×11) and (15×15×9) Monkhorst–Pack grids in the calculation concerning diamond and wz-GaN bulk crystals, respectively. During the geometry optimization step, forces acting on all atoms were no larger than 0.001 eV/Å. In the case of the supercell calculations performed for GaN crystal, a (6×6×1)*k*-point mesh was applied. The resulting, calculated lattice parameters of bulk crystals were the following: diamond: a=3.583967 Å, wz-GaN: a=3.215 Å, c/a=1.6236, in a good agreement with experimental data: 3.56683(1) Å (diamond) and a=3.190 Å, c/a=1.6269 (GaN) [12].

**Figure 1:**
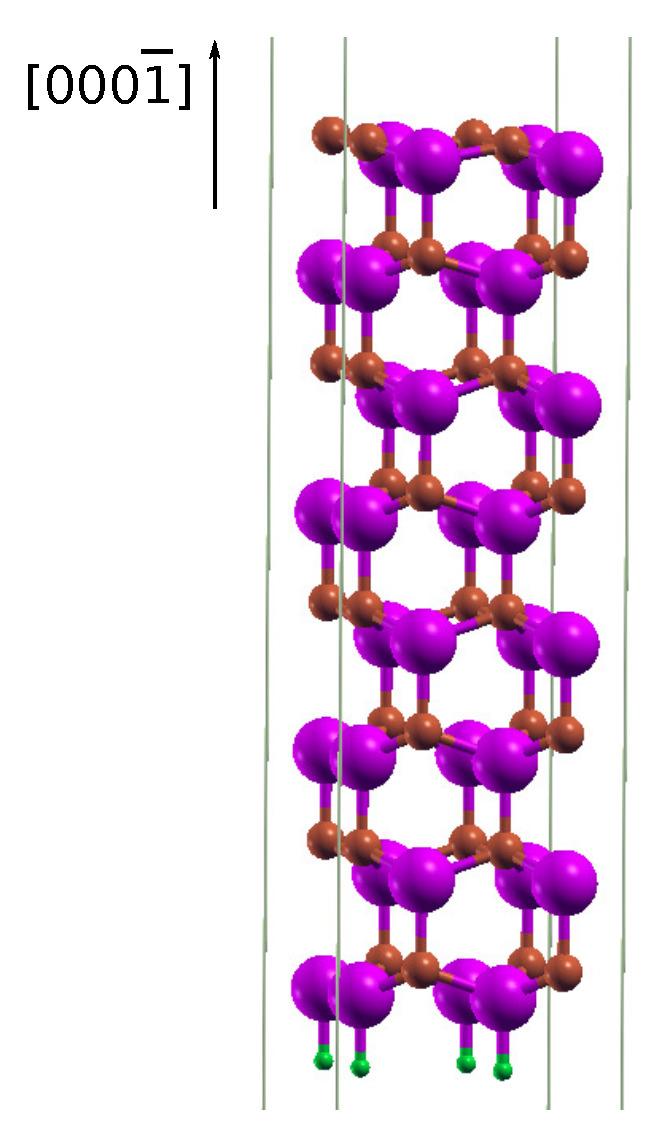
Exemplary slab model of the wz-GaN$\left(000\bar{1}\right)$ clean surface. Big violet and small brown balls represent Ga and N atoms, correspondingly. The smallest green balls denote saturating H pseudoatoms.

**Figure 2:**
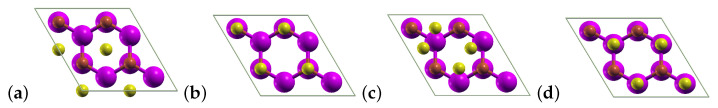
Adsorption sites indicated by yellow balls and defined in a 2×2 lateral unit cell representing GaN$\left(000\bar{1}\right)$ (N-terminated N-face). (**a**) H3. (**b**) On top. (**c**) Bridge. (**d**) T4.

Two models were utilized in the present calculations. Figure 1 presents an exemplary isolated slab having 8 double Ga–N layers, stacked along the hexagonal $\left[000\bar{1}\right]$ growth direction and used for the investigation of the carbon adsorption process on the GaN{0001} surfaces. The bottom surface of the slab, depending on the termination, was passivated by pseudo-hydrogen atoms of the fractional atomic numbers 0.75*e* or 1.25*e*. A 30 Å vacuum layer above the surface separated the slab from its periodic replicas. Three different sizes of the slab’s lateral unit cell were investigated, i.e., 1×1, 2×2, and 3×3, comprising 17, 68, and 153 atoms, respectively. Additionally, for the 2×2 surface, various coverages of C adsorbates were analyzed, 0.25 monolayer (ML), 0.5 ML, and 1 ML. The positions of all atoms of the slab were relaxed in the geometry optimization step of calculation with the imposed restriction on the forces to be less than 0.02 eV/Å. Figure 2 illustrates four possible types of high-symmetry adsorption sites $H_{3}$, on top, bridge, and $T_{4}$, presented in a lateral 2×2 unit cell. Simulations on carbon adsorption were performed using a Grimme correction scheme (DFT-D approach) ([35,36]). In this approach, London dispersion interactions were defined as the attractive part of the van der Waals-type interaction potential between atoms and molecules that were not directly bonded to each other. We applied a $C_{6}$ dispersion coefficient for the interacting C and N, as well as the C and Ga atoms.

**Figure 3:**
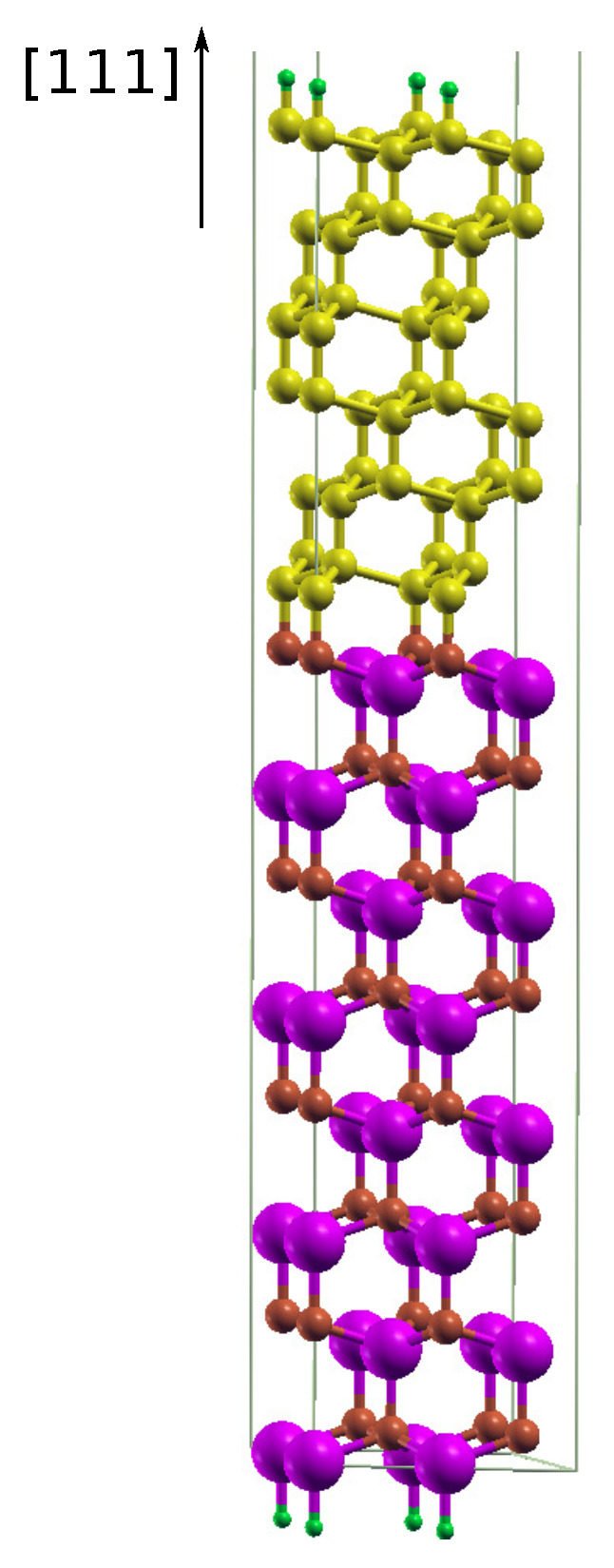
Slab model of the diamond–GaN heterostructure with the abrupt N-C interface type in the [111] growth direction. Large violet and small brown balls represent Ga and N atoms, correspondingly. Small yellow balls and tiny green balls represent C atoms and terminating H pseudoatoms, respectively.

Next, in the study on the influence of point detects on the stability of diamond–GaN interfaces, an isolated slab with 120 atoms and 2×2 lateral cell was used. Figure 3 displays the diamond–GaN heterointerface with the GaN substrate consisting of 8 Ga-N double layers arranged along $\left[000\bar{1}\right]$ and passivated by H-pseudoatoms. Depending on the substrate’s termination, two interface types between GaN and diamond were studied, i.e., N-C and Ga-C, referred to as the “abrupt” ones. The upper diamond part of the slab was built with 12 carbon monolayers, arranged along the [111] growth direction. The topmost surface of diamond is passivated by H atoms, and a vacuum layer of the same thickness of 30 Å is present at the top of slab. During the relaxation step, the forces acting on the atoms did not exceed 0.02 eV/Å.

Additionally, in the calculation concerning the heights of the migration energy barriers of certain point defects, a 5×5×3 supercell model of wz-GaN with 300 atoms in the growth direction [0001] was used. The calculation was performed by means of the nudged elastic band method [37,38,39].

## 3. Results

### 3.1. Carbon Adsorption on GaN{0001} Surfaces

The adsorption process was simulated in the following way. The carbon adsorbate was located above all studied on-surface adsorption sites defined on the GaN’s surface at distances 1.5–2 Å. After relaxation of the whole system, the most stable adsorption sites were found for all analyzed cases of coverage. The adsorption energy was calculated according to the formula:

(1) $$E_{\mathrm{ads}}=E_{C/\mathrm{surf}}-E_{\mathrm{surf}}-n\times E_{C},$$

where $E_{C/\mathrm{surf}}$ and $E_{\mathrm{surf}}$ describe the total energy of a slab with the adsorbed C atom and of a clean surface, correspondingly, *n* is the number of C adsorbate, and $E_{C}$ is the total energy of an isolated C atom.

#### 3.1.1. Ga-Terminated Surface

The adsorption process of C atoms on the GaN(0001) and GaN$\left(000\bar{1}\right)$ surfaces proceeded in a different way. In the case of the Ga-terminated Ga-face and the smallest coverage of 0.25 ML analyzed for the 2×2 surface cell, a carbon atom placed above either T4, H3, or on top sites adsorbed in the same site with the following adsorption energy $E_{ads}$: −7.603 eV/atom (T4), −7.407 eV/atom (H3), or −4.239 eV/atom (on top). However, when starting from the bridge site, the carbon atom migrated on the GaN(0001) surface and adsorbed finally with the minimal $E_{ads}=-7.696$ eV/atom in the T4 site, which is the most energetically stable one.

**Figure 4:**
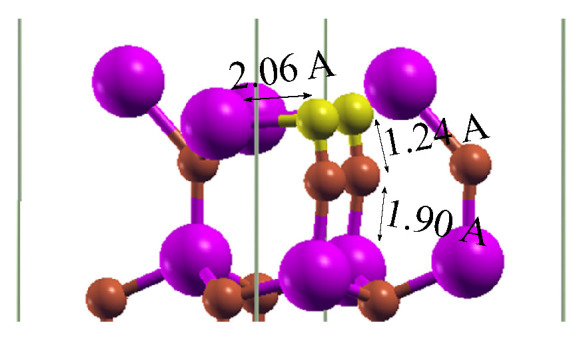
The most stable adsorption site in the case of 0.5 ML.

When the C coverage was increased up to 0.5 ML, a C atom starting both from H3 and on top sites adsorbed finally in the H3 site with the adsorption energy of −5.633 eV/atom and −5.655 eV/atom, respectively. The situation changed for other two positions. When the starting site was either T4 or the bridge one, then the final adsorption site was T4; however, C atoms were adsorbed under the topmost gallium layer (see Figure 4). As compared to previous sites, $E_{ads}$ was considerably lowered down to −7.480 eV/atom, which can be explained by the created bonds, i.e., the C atom bound in this case with the Ga and N atoms belonging to separate layers of GaN.

Table 1 presents the adsorption energies for C adsorbates on GaN(0001), obtained at the maximal coverage of 1 ML.

**Figure 5:**
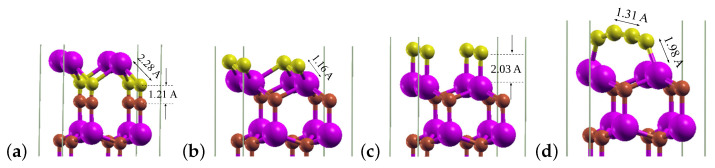
Final adsorption sites of carbon on GaN(0001). Coverage: 1 ML; adsorption is initiated in sites: (**a**) T4. (**b**) H3. (**c**) On top. (**d**) Bridge.

For the coverage of 1 ML, T4 was still the most energetically stable adsorption site, and C atoms adsorbed as before under the topmost Ga layer with $E_{ads}=-7.713$ eV/atom (compare Figure 5a). A C atom placed above the H3 and on top sites adsorbed in the same site with $E_{ads}$ equal to −4.273 eV/atom and −4.078 eV/atom, correspondingly (Figure 5b,c). Meanwhile, the bridge site turned out not to be a stable adsorption site, and carbon atoms placed there migrated on the surface and formed finally a chain of four atoms whose ends were bonded to Ga atoms (Figure 5d). It can be concluded that for this maximal coverage, only the H3 and on top sites can enable the formation of a single carbon monolayer on the GaN(0001) surface. In order to check this, we modeled the adsorption process of C atoms using a larger surface unit cell, i.e., a 3×3 one with nine carbon adsorbates. The study confirmed the above findings, with the only difference that the adsorption process initiated not only above T4, but also bridge sites, resulting in the final adsorption site T4; moreover, C atoms were adsorbed under the gallium layer.

**Figure 6:**
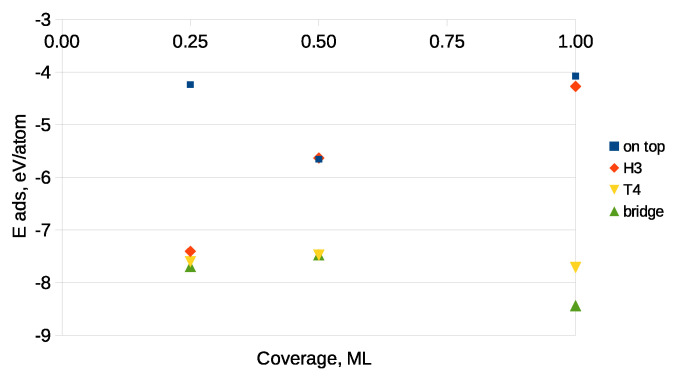
Adsorption energies vs. coverage on the Ga-terminated surface. Starting adsorption sites are shown in the legend panel.

Figure 6 presents the dependence of the adsorption energy on carbon coverage, established for a 2×2 surface cell on a Ga-terminated GaN surface. As can be seen, with the increasing coverage, the adsorption energies roughly became less negative due to an increased interaction between the adsorbates. This was the case for the H3 and on top sites, which enabled the formation of a single carbon monolayer. Additionally, lower (more negative) adsorption energies were observed for those sites for which either bonds of C with N atoms or C–C ones were possible.

#### 3.1.2. N-Terminated Surface

In the case of the N-terminated N-face and the smallest 0.25 ML coverage, a single C atom placed above the H3 site adsorbed there with the lowest adsorption energy $E_{ads}$ = −9.749 eV/atom. When the adsorption was initiated above the T4 and on top sites, the final adsorption site was on top with $E_{ads}$ equal to −9.420 eV/atom and −9.323 eV/atom, correspondingly. A carbon atom placed initially above the bridge position bound finally in a site shifted from the bridge, with $E_{ads}$ = −9.154 eV/atom. For the coverage of 0.5 ML and two carbon adsorbates, the adsorption process initiated above the T4, H3, and on top sites led to the final adsorption site shifting from on top, with $E_{ads}$ = −8.250 eV/atom. In the case of the process starting above the bridge site, one C atom was adsorbed in its vicinity, while the other one bound with a topmost N atom and two Ga atoms belonging to the lower lying gallium monolayer.

Table 2 presents the adsorption energies for C adsorbates on GaN$\left(000\bar{1}\right)$, obtained for the coverage of 1 ML.

**Figure 7:**
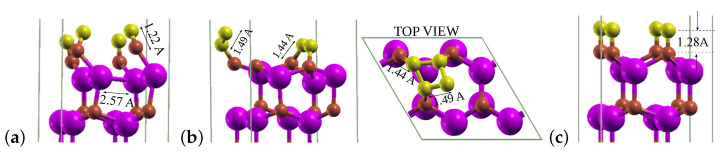
Final sites of C atoms on GaN$\left(000\bar{1}\right)$. Coverage: 1 ML; adsorption is initiated from sites: (**a**) T4 and H3. (**b**) Bridge. (**c**) On top.

For this maximal coverage of 1 ML, a reconstruction of the two uppermost nitrogen and gallium monolayers took place, which was induced by the adsorption of C atoms initiated either above the T4 or H3 sites (see Figure 7a). The required adsorption energy amounted to −7.205 eV/atom, and C atoms were bound to the top N atoms and slightly shifted from the on top sites. The adsorption initiated above the bridge site led to the formation of a cluster of 4 C atoms with the average C–C bond length of 1.49 Å (Figure 7b), and the process required $E_{ads}$ of −8.557 eV/atom. The only position that enabled the formation of a single carbon layer was for this coverage the on top one, and the corresponding $E_{ads}$ yielded −6.760 eV/atom (Figure 7c). The studies repeated for a larger 3×3 surface cell with nine carbon adsorbates led to analogous results, except for the starting site bridge, for which, in this case, the adsorption took place finally in a site shifted from on top.

**Figure 8:**
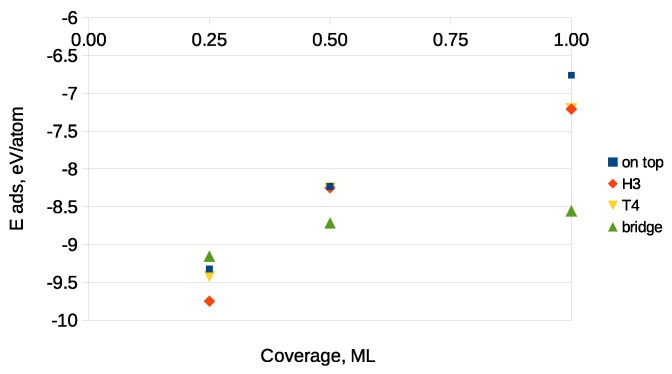
Adsorption energies vs. coverage on the N-terminated surface. Starting adsorption sites are shown in the legend panel.

The dependence of the adsorption energy versus coverage is shown in Figure 8.

**Figure 9:**
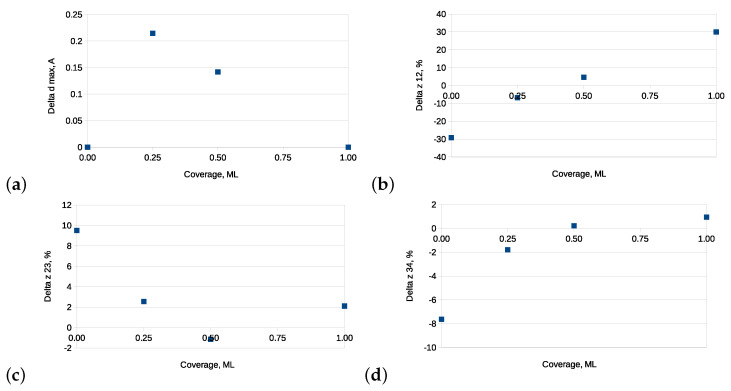
(**a**) Buckling of the topmost nitrogen monolayer, constituted based on the on top site. Relaxations of the topmost three interlayer spacings on the GaN$\left(000\bar{1}\right)$ surfaces vs. C coverage. (**b**) $\Delta z_{12}$. (**c**) $\Delta z_{23}$. (**d**) $\Delta z_{34}$.

As can be seen, with the increasing carbon coverage, the adsorption energies became less negative, which presented a typical behavior caused by the enhanced interaction between the adsorbates. Moreover, the adsorption energies of the C atom on the GaN$\left(000\bar{1}\right)$ surface were about 2 eV/atom larger (more negative) as compared to those of GaN(0001), what indicated that the deposition process of carbon took place preferentially on GaN$\left(000\bar{1}\right)$. Moreover, the only adsorption site enabling the formation of a single carbon monolayer on the GaN$\left(000\bar{1}\right)$ surface was the on top one. Hence, it was reasonable to analyze here an influence of the adsorption process on the GaN’s substrate morphology. Figure 9a displays the buckling of the topmost nitrogen monolayer, established for the on top adsorption site of carbon. As a measure of buckling, we assumed here the maximal difference $\Delta d_{max}$ between the *z*-th positions of the topmost nitrogen atoms expressed in Å. As can be traced from Figure 9a, the buckling varied with coverage, assuming a maximal value for 0.25 ML. When the GaN$\left(000\bar{1}\right)$ surface was completely covered with carbon adsorbates, the buckling vanished, which corroborated the presence of a flat pre-adsorbed carbon monolayer. The adsorption process additionally changed the atomic structure of the GaN$\left(000\bar{1}\right)$ substrate. This can be observed in the plots of the interlayer distance difference $\Delta z_{ij}$ of the three topmost GaN$\left(000\bar{1}\right)$ layers presented in Figure 9b,c. $\Delta z_{ij}$ denotes a difference in spacing between the *i*-th and *j*-th substrate’s layer with respect to that of the clean surface before relaxation. The values of $\Delta z_{ij}$ changed roughly linearly as the coverage increased, and the tendency of reducing the effect of relaxation could be additionally observed for the second and third interlayer spacing ($\Delta z_{23}$, $\Delta z_{34}$). In the case of the first interlayer spacing $\Delta z_{12}$, changes induced by carbon adsorption reached up to 30% for the full coverage.

### 3.2. Diamond–GaN Interface

Based on the above obtained results, two types of diamond–GaN heterointerfaces were constructed, as described in Section 2. The main issue related to the model of the interface was the way of joining interface atoms, i.e., C and N, as well as C and Ga. Since the modeling of C adsorption revealed the on top adsorption site to be the most energetically stable one on the GaN$\left(000\bar{1}\right)$ surface, the diamond part was overlaid on this site to achieve the N-C interface type. As a result, at the N-C interface type presented in Figure 3, long bonds joined tetrahedrally coordinated N and C atoms of both materials. In the case of the Ga-terminated GaN surface, we chose the same way of joining Ga and C interface atoms, by overlaying the on top site with the diamond structure, thus resulting in the Ga-C interface type. In this way, the energetic stability of both interface types could be directly compared. The on top adsorption site on the GaN(0001) surface was slightly less energetically stable than H3 (both of them enabled the formation of a carbon monolayer); therefore, it could be tested whether it was possible to enhance the energetic stability of the whole system utilizing a particular reconstruction.

**Figure 10:**
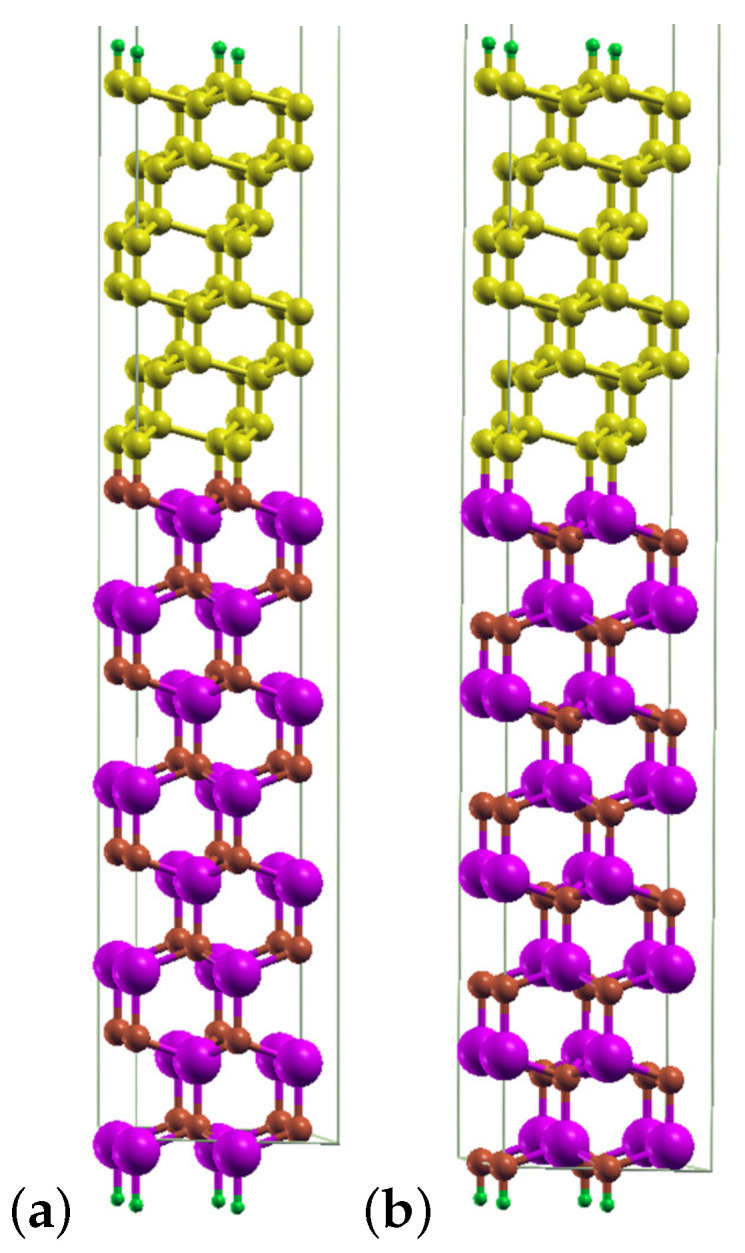
A relaxed diamond–GaN heterostructure with the (**a**) N-C abrupt interface type and (**b**) Ga-C abrupt interface type.

After the relaxation of the whole system, the diamond structure was preserved on the GaN substrate; see Figure 10a,b. In this figure, the diamond part is strained to the lateral unit cell of GaN. The heterostructure with the abrupt N-C interface type exhibited N–C bonds with a length of about 1.48 Å. C–C bonds in diamond in the [111] direction had an average length of 1.50 Å, while those joining carbon monolayers that were perpendicular to [111] exhibited an average length of 1.93 Å (Figure 10a). Figure 10b presents a relaxed structure of the diamond–GaN heterointerface with the abrupt Ga-C interface type. The average length of Ga–C long bonds amounted to 2.02 Å. The C–C average bond length in the [111] direction was 1.48 Å and 1.93 Å in the direction perpendicular to [111]. These differences in bond length corroborated the presence of the strained diamond structure in both cases.

**Figure 11:**
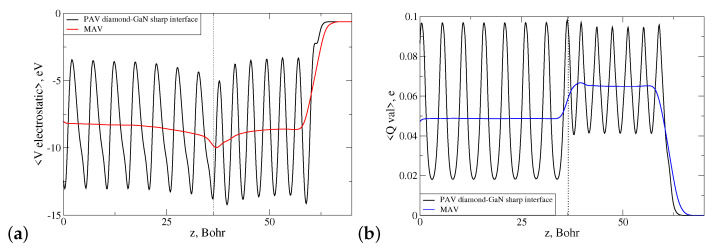
Laterally (PAV) and macroscopically (MAV) averaged profiles of the (**a**) electrostatic potential and (**b**) valence electron charge density of the N-C abrupt diamond–GaN interface type. A dotted line points to the average z-th position of the topmost N atoms.

Both interface types were influenced by an internal electric field arising due to the presence of spontaneous and piezoelectric polarization in the wz-GaN material. Due to the different substrate polarities, these fields had opposite directions in both structures. Moreover, due to the different valencies of the species at the interface, long N-C and Ga-C bonds were oversaturated and undersaturated with electrons, respectively. This means that there was a portion of uncompensated electron charge present in the lateral cell of each interface as a monopole. These two factors influenced the overall energetic stability of the discussed diamond–GaN interface. To investigate this issue, a profile of the electrostatic potential $\langle V_{el}\rangle$ was computed as a function of the heterostructure height *z* for both heterostructures, and next, its macroscopic average in-plane xy was found, as well. Figure 11a presents the course of both functions denoted as PAV and MAV for the abrupt N-C interface type. The function $\langle V_{el}\rangle$ exhibited a negative slope in the range z=0−36.43 Bohr, which corresponded to the height of the GaN substrate. This behavior indicated the presence of a nonzero electric field present in the system. As can be observed in Figure 11a, this field penetrated the diamond part of the heterostructure for z>36.43 Bohr, which manifested itself by the increasing, positive slope of the macroscopically averaged $\langle V_{el}\rangle$ function denoted by red color (MAV). It should be noted here that in present calculation, a slab dipole correction was used to compensate the electric field in the vacuum area caused by the dipole moment of the system [40].

Additionally, we computed the profile of the valence electron charge density of the diamond–GaN heterointerface, averaged laterally and macroscopically, which is presented in Figure 11b. As can be seen, in the interface area, a small, localized pileup of the uncompensated electron charge was present, which is discussed in Section 4.1.

**Figure 12:**
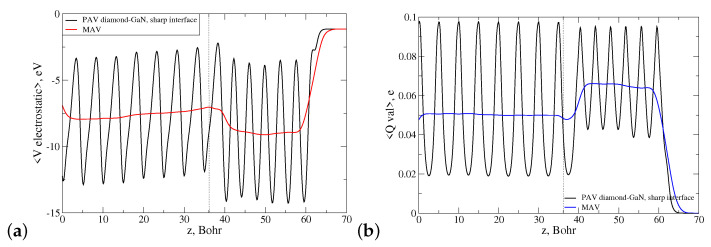
Laterally (PAV) and macroscopically (MAV) averaged profiles of the (**a**) electrostatic potential and (**b**) valence electron charge density of the Ga-C abrupt diamond–GaN interface type. A dotted line points to the average z-th position of the topmost Ga atoms.

An analogous situation concerning the presence of the electric field and uncompensated electron charge took place in the case of the abrupt Ga-C interface type. As follows from Figure 12a, the built-in internal electric field in the wz-GaN substrate possessed the inverse sign to that of the N-C interface, and its presence was manifested by the positive slope of the macroscopic average of electrostatic potential. This field first entered the monolayers of the diamond structure, situated at z>36.09 Bohr. The discussed profile of $\langle V_{el}\rangle$ was accompanied by the computed profile of laterally and macroscopically averaged valence electron charge density, showing again an accumulation of uncompensated charge in the interface area, presented in Figure 12b.

In summary, we demonstrated two main factors that influenced the mechanical and energetic stability of the diamond–GaN interfaces. In the next section, we discuss possible ways to improve this stability.

## 4. Discussion

### 4.1. Charge Compensation

**Figure 13:**
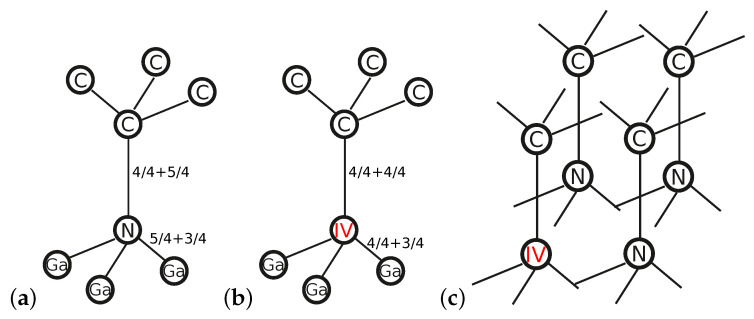
Scheme of long tetrahedral bonds with fractions of the valence electron charge at the (**a**) abrupt N-C diamond–GaN interface type. (**b**) Supplemental configuration with a IV-valency atom substituting the N atom. (**c**) Reconstructed N-C diamond–GaN interface type with a IV-valency substitutional atom in a surface 2×2 unit cell.

All three technologies of GaN-on-diamond fabrication presented in the Introduction require the creation of bonding between species of different valencies. In the case of the abrupt N-C diamond–GaN interface type presented in Figure 10a, the corresponding tetrahedral configuration in the vicinity of long interface bonds is depicted schematically in Figure 13a.

As can be seen, a V-valency N atom donated to each bond 5/4 of the electron charge. Since it was joined with the IV-valency C atom from the diamond material, there was an excess of 1/4e per each long bond between N and C, as compared with the charge of the electron pair. As a result, there was an excess of one valence electron in a 2×2 lateral unit cell encompassing four such bonds, which led to a certain amount of uncompensated electron charge at the interface. By analogy, it can be proven that there was a deficit of one valence electron in the 2×2 lateral unit cell of the abrupt Ga-C interface type presented in Figure 10b. This uncompensated electron charge can be identified at the interface areas in Figure 11b and Figure 12b.

A natural way to improve the energetic stability of N-C and Ga-C diamond–GaN interface types is to compensate as a first step the electron charge present at the interface. This can be done by means of appropriate substitutional dopants introduced at the interface area. Note that the substitution of a V-valency N atom by a IV-valency one in one tetrahedral configuration depicted in Figure 13b would lead to a deficiency of 3/4e in its neighborhood. Therefore, in the case of a lateral 2×2 unit cell comprising tetrahedrally coordinated atoms with one reconstructed bond, as shown in Figure 13c, the requirement of charge neutrality was fulfilled. The corresponding reconstruction pattern within the lateral unit cell of the N-C interface type was $3N+1X_{N}$, where $X_{N}$ stands for a IV-valency atom substituting nitrogen.

Analogously, it can be checked that in the case of the Ga-C interface type, substitution of a III-valency Ga atom by a IV-valency one in the configuration corresponding to that of Figure 13b led to an abundance of 3/4e in the neighborhood of the substituting atom. The resulting configuration of four tetrahedrally coordinated bonds with one substitutional atom present in the 2×2 lateral unit cell would guarantee valence charge neutrality within the analyzed interface’s unit cell. Hence, the corresponding reconstruction pattern within the lateral cell of the Ga-C interface type was $3Ga+1X_{Ga}$, where $X_{Ga}$ again stands for a IV-valency atom substituting, this time, a Ga atom.

Inasmuch as the proposed reconstruction patterns involve the topmost GaN substrate’s layer, natural candidates for substitutional atoms are Si and C. The GaN material utilized in HEMTs is usually epitaxially grown on Si(111) or silicon carbide substrate layers [5,41]. These layers are either mechanically removed or chemically etched in GaN-on-diamond bonding technology [5,20,21,22]. However, in order to improve the mechanical/energetic stability of diamond–GaN interfaces, the desired substitutional atom should not introduce additional strain to the system. Hence, another aspect should be taken into account, i.e., an appropriate relation between the atomic radius of $r_{host}$ and $r_{dopant}$. Since $|r_{N}-r_{C}|=0.07$ Å, $|r_{N}-r_{Si}|=0.47$ Å, $|r_{Ga}-r_{C}|=0.49$ Å, and $|r_{Ga}-r_{Si}|=0.09$ Å [42], the assumed reconstruction patterns should be as follows: $3N+1C_{N}$ in the case of the N-C interface type and $3Ga+1Si_{Ga}$ in the case of the Ga-C diamond–GaN interface type.

### 4.2. Migration of Point Defects in Bulk wz-GaN Crystal

Before carrying out the proposed reconstruction in the topmost GaN substrate layer, we calculated the formation energies and heights of the energy barriers encountered by certain substitutional dopants in wz-GaN crystal, as explained in Section 2. It should be noted here that an extensive analysis concerning defects in GaN can be found in [43,44,45,46]. As for carbon-related defects, Kyrtsos el al. concentrated mainly on C interstitial dopants, as well as their complexes [44]. Interestingly, in the case of nitrogen interstitials, $N_{i}$ and $C_{N}$ could cause the creation of $C_{i}$, and the related migration energy barrier was reported to be 2.3 eV. In our analysis, we concentrated on the vacancy-mediated mechanism of dopant diffusion and studied the migration of substitutional dopants both in the hexagonal *c*-axis direction and perpendicular to it, the *a*-axis direction. This choice allowed gaining the overall information about the possibility of a given reconstruction to occur in the GaN substrate. Table 3 presents the computed formation energies and heights of the migration energy barriers of the selected single substitutional dopants, as well as their complexes.

As can be seen from Table 3, the heights of the migration energy barriers were lower for substitutional dopants forming complexes with vacancies than those of single substitutional dopants; the former reached up to ∼4.6 eV. However, high formation energies indicated that some of them (e.g., $Si_{N}+V_{N}$) could be formed only in a harsh environment. In contrast, a diffusion of a charged $Si_{N}+V_{N}^{3+}$ complex could take place spontaneously after overcoming the energy barrier of 0.3 eV or 0.9 eV, depending on the diffusion direction.

### 4.3. Influence of Point Defects on the Stability of the Diamond–GaN Interfaces

**Figure 14:**
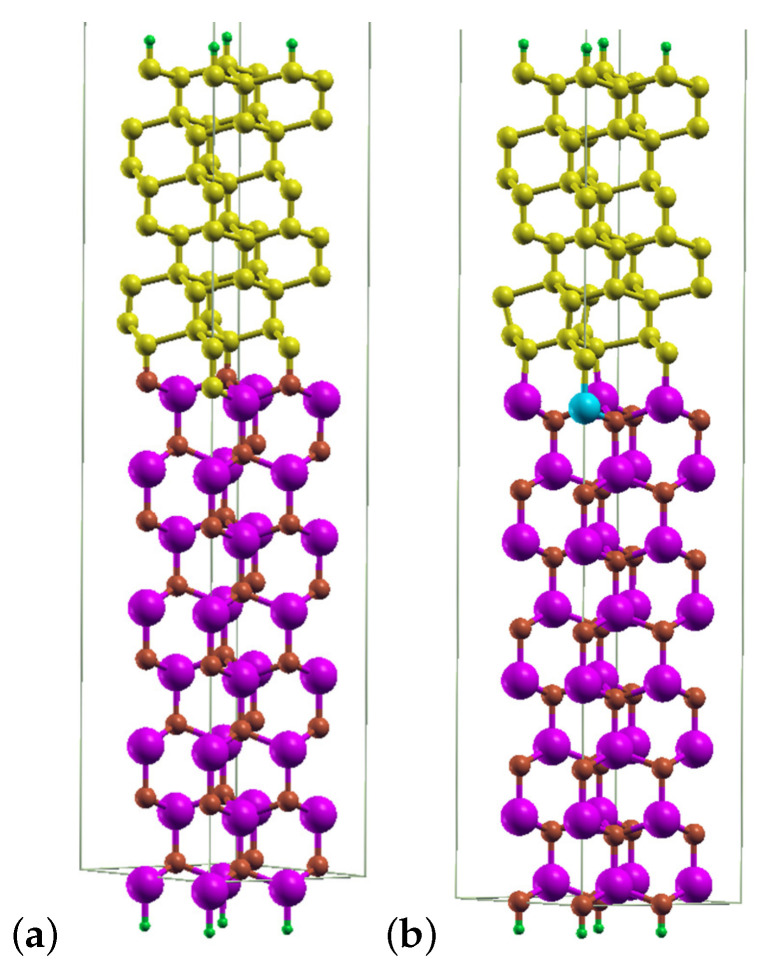
Perspective view at the diamond–GaN heterostructure with the reconstructed interface according to the pattern (**a**) $3N+1C_{N}$ and (**b**) $3Ga+1Si_{Ga}$. The large blue ball represents the Si atom.

Next, $C_{N}$ and $Si_{Ga}$ substitutional dopants were introduced into the interface lateral 2×2 unit cell of the GaN substrate according to the reconstruction patterns $3N+1C_{N}$ and $3Ga+1Si_{Ga}$ predicted in Section 4.1. The structures of the relaxed, reconstructed heterointerfaces are shown in Figure 14a,b. Both reconstructions changed the length of the interface bonds. In the case of the reconstructed N-C interface type, the C–C bond length was 1.52 Å and three other N–C bonds possessed an average length of 1.48 Å. In the case of the Ga-C interface type, the Si–C bond length was 1.86 Å, and the average Ga–C bond length was 1.99 Å.

**Figure 15:**
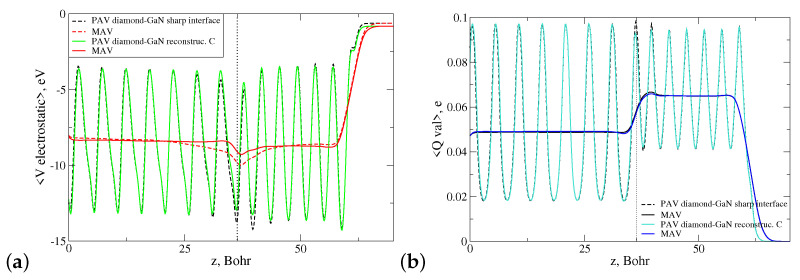
Course of laterally (PAV) and macroscopically (MAV) averaged profiles of the (**a**) electrostatic potential and (**b**) valence electron charge density in the diamond–GaN heterostructure with the N-C abrupt and reconstructed interface types. The applied reconstruction pattern is $3N+1C_{N}$. A dotted line points to the average z-th position of the topmost N atoms.

Figure 15a displays the dependence of the laterally (PAV) and macroscopically averaged (MAV) electrostatic potentials $\langle V_{el}\rangle$ of the abrupt and reconstructed N-C interface (pattern $3N+1C_{N}$) versus heterostructure height *z*.

As can be observed, the slope of macroscopic average of $\langle V_{el}\rangle$ (MAV) in the interface area changed considerably for the reconstructed interface, and it became a horizontal line (red solid line in Figure 15a in both parts of the heterointerface. We interpreted this behavior in such a way that the $C_{N}$ dopant reduced the electric field entering the diamond part of the diamond–GaN heterointerface. The corresponding profile of the MAV function for valence electron charge density presented in Figure 15b changed only slightly, as the reconstruction within the lateral cell concerned theoretically the exchange of one valence electron.

**Figure 16:**
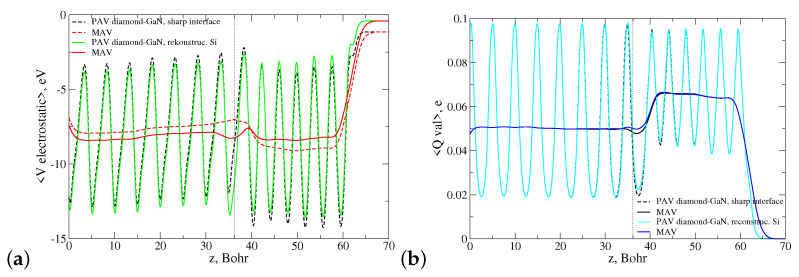
Course of laterally (PAV) and macroscopically (MAV) averaged profiles of the (**a**) electrostatic potential and (**b**) valence electron charge density in the diamond–GaN heterostructure with the Ga-C abrupt and reconstructed interface types. The applied reconstruction pattern is $3Ga+1Si_{Ga}$. A dotted line points to the average z-th position of the topmost Ga atoms.

Next, we computed the profiles of $\langle V_{el}\rangle$ and $\langle Q_{val}\rangle$ for the Ga-C interface type reconstructed according to the pattern $3Ga+1Si_{Ga}$. The course of both functions is shown in Figure 16. This time, the applied reconstruction evoked essential changes in the relaxed atomic positions and, as a consequence, in the course of the electrostatic potential within the structure. Nevertheless, the effect of diminishing the electric field in the diamond monolayers next to the interface was still observed. Interestingly, the changes evoked by the reconstruction in the course of the macroscopically averaged valence electron charge density were more pronounced than in the case of the reconstructed N-C interface type. They revealed the effect of valence charge compensation in the vicinity of the interface, i.e., a transition from the monopole-like to dipole-like shape.

Finally, we tested two other possible reconstruction patterns induced by dopant atoms $Si_{N}$ and $C_{Ga}$ that ensured the charge neutrality of the respective reconstructed lateral cell, whose radius however did not match the relation $r_{host}\approx r_{dopant}$. In the case of the N-C diamond–GaN interface type, this was the pattern $3N+1Si_{N}$, and in the case of the Ga-C type, the pattern was $3Ga+1C_{Ga}$. Additionally, to examine the issue of the energetic stability of the studied interfaces, we computed the energy gain per one primitive lateral cell of the system evoked by reconstruction, by means of the formula [47,48]:

(2) $$\Delta H=\frac{1}{2\times 2}\left(E_{tot}^{slab\;abr.}-E_{tot}^{slab\;recon.}+\mu_{recon.atom}-\mu_{orig.atom}\right),$$

where ${\left(2\times 2\right)}^{-1}$ is the surface of the chosen lateral unit cell in units of bulk lateral cell, $E_{tot}^{slab\;abr.}$ and $E_{tot}^{slab\;recon.}$ represent the total energy of the slabs with the abrupt and reconstructed interfaces, correspondingly, and $\mu_{recon.atom}$ denotes the chemical potential of each dopant atom that substituted an original atom with chemical potential $\mu_{orig.atom}$.

Table 4 displays the computed values of ΔH for all studied interface types and all discussed reconstruction patterns.

As follows from Equation (2), a positive sign of ΔH is related to the energy gain of the system, induced by a substitutional atom present in the topmost GaN substrate’s layer. As follows from Table 4, only the reconstructions evoked by the presence of substitutional atom that holds the relation $r_{host}\approx r_{dopant}$ were energetically favorable. These were in particular the $3N+1C_{N}$ and $3Ga+1Si_{Ga}$ ones for the N-C and Ga-C diamond–GaN interface types, respectively. The maximum energy gain per one lateral unit cell was ∼0.53 eV/cell, and it was observed for the N-C reconstructed interface type, according to the pattern $3N+1C_{N}$.

This behavior can be explained by analyzing the strain of the system induced by the presence of substitutional dopants. The strain manifested itself by the changes of the system’s internal pressure ΔP, presented in Table 4. Both reconstructions improving the energetic stability of the studied interfaces were related either with a small decrease in the total pressure of the system or with a small increase of this quantity, of the order of 0.8 kBar. The reconstructions that were unfavorable energetically ($3N+1Si_{N}$ and $3Ga+1C_{Ga}$) evoked larger changes of the system’s total pressure. An analogous situation took place in the case of the reconstructed diamond–AlN interfaces [26]. Both the wz-GaN and wz-AlN materials exhibited the built-in electric field originating from the spontaneous and piezoelectric polarizations, as well as a lattice mismatch with diamond. In spite of the spontaneous polarization in wz-AlN being about three times larger than that of wz-GaN, the effect of controlling the interface stability remained on the same level, i.e., the maximal energy gain in the case of the reconstructed diamond–AlN interface was in the range 0.14–0.46 eV.

**Figure 17:**
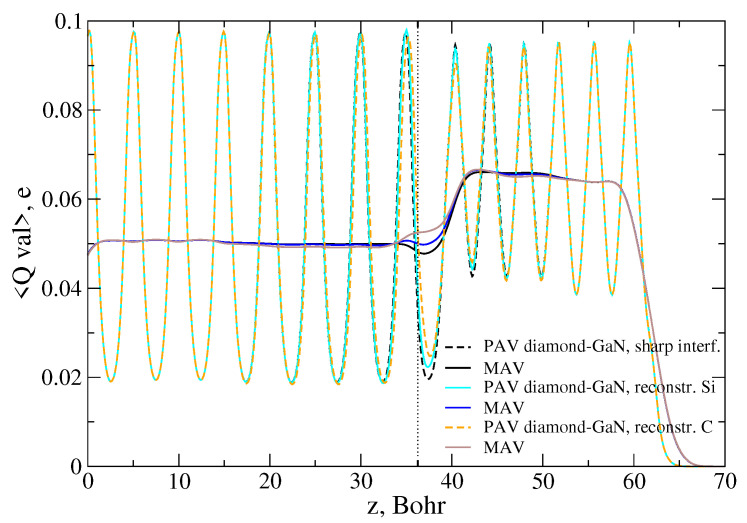
Comparison of the macroscopically (MAV) averaged profiles of the valence electron charge density in the diamond–GaN heterostructure with the Ga-C abrupt and reconstructed interface types, according to patterns $3Ga+1Si_{Ga}$ and $3Ga+1C_{Ga}$. A dotted line points to the average z-th position of the topmost Ga atoms.

Therefore, the requirement of charge neutrality within a surface unit cell of the diamond–GaN interface was not enough to warrant the related reconstruction to be energetically favorable. Moreover, our calculation showed that, e.g., in the case of the Ga-C interface type, the predicted charge transfer connected to an exchange of the III-valency Ga atom with a IV-valency one according to the patterns $3Ga+1C_{Ga}$ and $3Ga+1Si_{Ga}$ was not the same. This statement can be illustrated by the common plot of the macroscopically averaged profiles of the valence electron charge density in the diamond–GaN heterostructure, presented in Figure 17.

Both reconstruction types presented the effect of valence charge compensation in the vicinity of the Ga-C interface, i.e., a transition from the monopole-like to dipole-like shape; however, the related charge transfer was not identical. It was conditioned by the value of the electronegativity of the C and Si species.

The presented reconstruction patterns could be compared with experimental results concerning the fabrication of the GaN/diamond heterointerface via direct bonding. In particular, the energy dispersive spectroscopy (EDS) mapping revealed in the as-bonded GaN/diamond heterointerface fabricated by a surface-activated bonding (SAB) method [23] the presence of an intermediate layer with the C, Ga, O, and N atoms. After annealing in 700 ${}^{\circ }$C and 1000 ${}^{\circ }$C, the thickness of the intermediate layer decreased down to 1.5 nm, and the C atoms diffused into the GaN substrate adjacent to the intermediate layer. The diffusion depth was determined to be greater than 4 nm.

In turn, in the experiments on the diamond growth on the GaN covered with a SiN dielectric layer [16], it was concluded that a few-nanometer-thick Si-rich SiN layer could convert to SiC before diamond nucleation, and Si–C bonding during diamond nucleation was beneficial to the strong adhesion of the obtained diamond films.

## 5. Conclusions

We investigated the adsorption process of carbon atoms on GaN{0001} surfaces, as well as the influence of selected substitutional dopants on the energetic stability of diamond–GaN interfaces.

In particular, we showed that the only stable adsorption site on the N-terminated GaN surface that enabled the formation of a flat carbon monolayer was the on top one. In the case of the Ga-terminated surface, two such positions, on top and H3, were revealed; however, the corresponding adsorption energies were smaller than that of the N-terminated surface. Next, we discussed the possibility of controlling the diamond–GaN interface’s stability by means of certain substitutional dopants and reconstruction patterns. For this purpose, a model of the diamond–GaN heterojunction was constructed with the Ga-C or N-C interface type, justified in the results concerning carbon adsorption on clean GaN{0001} surfaces. By means of the calculated profiles of the laterally averaged electrostatic potential and its macroscopic average, we demonstrated the presence of a built-in electric field in GaN that affected the diamond part of the heterointerface, as well as of a localized pileup of electron charge in the interface area. Next, taking into account the requirement of charge neutrality within a lateral cell of the interface, we demonstrated that substitutional $C_{N}$ and $Si_{Ga}$ atoms introduced to the topmost nitrogen and gallium layers of the respective abrupt N-C and Ga-C interface types enhanced the energetic stability of the whole system. The presence of these dopants reduced the electric field penetrating the diamond part of the abrupt heterojunction and compensated the valence electron charge in a lateral unit cell of the diamond–GaN reconstructed interface. Additionally, we showed that amongst the substitutional dopants $C_{N}$, $Si_{Ga}$, $Si_{N}$, and $C_{Ga}$ that are common in GaN-on-diamond bonding technology, only $C_{N}$ and $Si_{Ga}$ fulfilled both the requirement of charge neutrality of the reconstructed interface lateral cell and of reducing the strain present at the heterointerface. Finally, we estimated the heights of the energy barriers that should be overcome during the vacancy-mediated diffusion of these dopants in bulk GaN crystals.

The results obtained in this paper contribute to GaN-on-diamond technology with respect to the choice of the appropriate interfacial layer during heteroepitaxial diamond growth on GaN or the preparation of a GaN substrate for direct bonding with diamond.

## Figures and Tables

**Table 1:** Adsorption energies for C adsorbates on the GaN(0001) surface. Coverage 1 ML, 2×2 lateral cell.

Starting Site	$E_{\mathrm{ads}},$ eV/atom	Final Site
T4	−7.713	T4 under Ga layer
H3	−4.273	H3 (ML)
on top	−4.078	on top (ML)
bridge	−8.441	chain of 4 atoms

**Table 2:** Adsorption energies for C adsorbates on the GaN$\left(000\bar{1}\right)$ surface. Coverage 1 ML; 2×2 lateral cell.

Starting Site	$E_{\mathrm{ads}},$ eV/atom	Final Site
T4	−7.202	shifted from on top
H3	−7.208	shifted from on top
on top	−6.760	on top (ML)
bridge	−8.557	cluster of 4 atoms

**Table 3:** Heights of the migration energy barriers of selected substitutional dopants in wz-GaN crystal. N-rich growth conditions are assumed.

Defect Type	Charge State	$E_{\mathrm{formation}}$, eV	$E_{\mathrm{barrier}}$, eV
$C_{N}+V_{N}$	0	5.70	*c: 4.09*
			*a: 3.36*
	+3	5.57	*c: 2.26*
			*a: 1.67*
$Si_{Ga}+V_{Ga}$	0	9.31	*c: 4.58*
			*a: 3.70*
	−3	5.40	*c: 3.94*
			*a: 3.28*
$Si_{N}+V_{N}$	0	11.13	*c: 2.54*
			*a: 1.89*
	+3	9.33	*c: 0.93*
			*a: 0.30*
$Si_{N}$	0	9.37	*c: 5.72*
			*a: 5.71*
$Si_{Ga}$	0	3.01	*c: 12.27*
			*a: 12.02*

**Table 4:** Energy gain due to reconstruction in the topmost GaN substrate’s layers.

Interface Type	Reconstruction Pattern	ΔH, eV/cell	ΔP, kBar	$|r_{\mathrm{host}}-r_{\mathrm{dopant}}|$, Å
*N*–*C*	$3N+1C_{N}$	0.530	−3.276	0.07
*N*–*C*	$3N+1Si_{N}$	−0.261	−18.890	0.47
Ga–*C*	$3Ga+1C_{Ga}$	−0.634	4.258	0.49
Ga–*C*	$3Ga+1Si_{Ga}$	0.460	0.735	0.09

## Data Availability

The data presented in this study are available on request from the corresponding author.
